# Supplementary material for: Transcriptomic Insights into Mechanisms of Early Seed Maturation in the Garden Pea (Pisum sativum L.)
Source: Cells. 2020 Mar 23;9(3):779. doi: 10.3390/cells9030779 (PMC7140803; doi:10.3390/cells9030779)

Sprint−2(10) vs Zhewa

Global Summary

%DE = 0.9

genes with fdr < 0.2 = 1881 (929 + / 952 −)

genes with fdr < 0.1 = 1698 (839 + / 859 −)

genes with fdr < 0.05 = 1610 (793 + / 817 −)

genes with fdr < 0.01 = 1197 (585 + / 612 −)

genes in genesets = 0

<FC> = 0

<t−score> = −1.95

<p−value> = 0

<fdr> = 0.1

Global Genelist

Rank	ID	log(FC)	p−value	fdr	Metagene	Description
1	TRINITY_DN1	−0.03	2e−12	0.0000000[illegible]	[illegible] x 11	
2	TRINITY_DN1	−0.03	6e−11	0.0000000[illegible]	[illegible] x 16	
3	TRINITY_DN1	−0.01	1e−10	0.0000000[illegible]	[illegible] x 18	
4	TRINITY_DN1	−0.04	2e−10	0.0000000[illegible]	[illegible] x 19	
5	TRINITY_DN8	−0.54	4e−10	0.0000000[illegible]	[illegible] x 25	
6	TRINITY_DN1	−0.01	7e−10	0.0000001[illegible]	[illegible] x 10	
7	TRINITY_DN1	−0.01	1e−09	0.0000001[illegible]	[illegible] x 13	
8	TRINITY_DN2	−1.5	2e−09	0.0000003[illegible]	[illegible] x 30	
9	TRINITY_DN1	−0.01	2e−09	0.0000001[illegible]	[illegible] x 22	
10	TRINITY_DN2	−0.02	2e−09	0.0000003[illegible]	[illegible] x 14	
11	TRINITY_DN1	−0.01	6e−09	0.0000003[illegible]	[illegible] x 13	
12	TRINITY_DN1	−2.12	6e−09	0.0000003[illegible]	[illegible] x 30	
13	TRINITY_DN8	−0.03	7e−09	0.0000004[illegible]	[illegible] x 18	
14	TRINITY_DN9	−2.23	9e−09	0.0000004[illegible]	[illegible] x 30	
15	TRINITY_DN1	−0.14	1e−08	0.0000004[illegible]	[illegible] x 1	
16	TRINITY_DN1	−0.02	1e−08	0.0000004[illegible]	[illegible] x 17	
17	TRINITY_DN6	−2.71	1e−08	0.0000004[illegible]	[illegible] x 30	
18	TRINITY_DN5	0.53	2e−08	0.0000006[illegible]	[illegible] x 1	
19	TRINITY_DN1	−0.01	2e−08	0.0000006[illegible]	[illegible] x 15	
20	TRINITY_DN1	−0.26	2e−08	0.0000009[illegible]	[illegible] x 26	

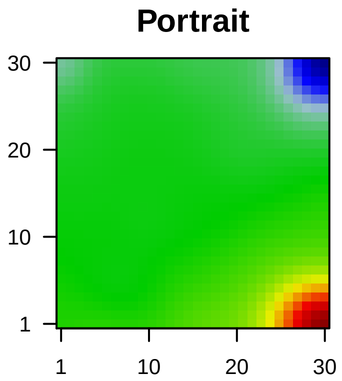

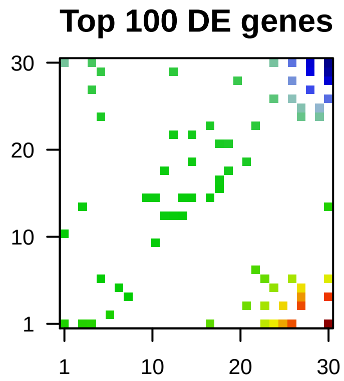

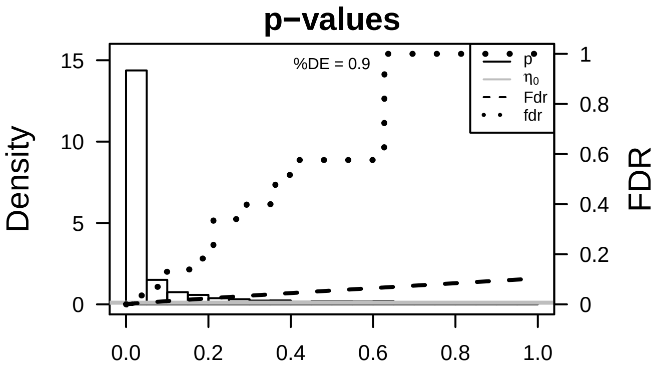

Supplement: Supplementary file 1 [file cells-09-00779-s001.zip › Supplementary materials/FileS4.zip!/FileS3/TFs - Results/Summary Sheets - Differences/Reports/Sprint.2.10..vs.Zhewan.1.25..pdf]
